# Hunter-Gatherer Energetics and Human Obesity

**DOI:** 10.1371/journal.pone.0040503

**Published:** 2012-07-25

**Authors:** Herman Pontzer, David A. Raichlen, Brian M. Wood, Audax Z. P. Mabulla, Susan B. Racette, Frank W. Marlowe

**Affiliations:** 1 Department of Anthropology, Hunter College, New York, New York, United States of America; 2 New York Consortium in Evolutionary Primatology, New York, New York, United States of America; 3 School of Anthropology, University of Arizona, Tucson, Arizona, United States of America; 4 Department of Anthropology, Yale University, New Haven, Connecticut, United States of America; 5 Department of Archeology, University of Dar es Salaam, Dar es Salaam, Tanzania; 6 Washington University School of Medicine, St. Louis, Missouri, United States of America; 7 Division of Biological Anthropology, University of Cambridge, Cambridge, United Kingdom; University of California, San Francisco, United States of America

## Abstract

Western lifestyles differ markedly from those of our hunter-gatherer ancestors, and these differences in diet and activity level are often implicated in the global obesity pandemic. However, few physiological data for hunter-gatherer populations are available to test these models of obesity. In this study, we used the doubly-labeled water method to measure total daily energy expenditure (kCal/day) in Hadza hunter-gatherers to test whether foragers expend more energy each day than their Western counterparts. As expected, physical activity level, PAL, was greater among Hadza foragers than among Westerners. Nonetheless, average daily energy expenditure of traditional Hadza foragers was no different than that of Westerners after controlling for body size. The metabolic cost of walking (kcal kg^−1^ m^−1^) and resting (kcal kg^−1^ s^−1^) were also similar among Hadza and Western groups. The similarity in metabolic rates across a broad range of cultures challenges current models of obesity suggesting that Western lifestyles lead to decreased energy expenditure. We hypothesize that human daily energy expenditure may be an evolved physiological trait largely independent of cultural differences.

## Introduction

By 2015, nearly one in every three people worldwide is projected to be overweight, and one in ten is expected to be obese [1]. The attendant health risks of being overweight or obese, including Type 2 diabetes, cardiovascular disease, and certain cancers, are well known [1]. The proximate cause of weight gain is energy imbalance, with food energy intake (kCal/day) exceeding total energy expenditure (kCal/day), but the societal causes of the global obesity pandemic remain a focus of debate [1]–[7]. Generally, rising obesity incidence is thought to result from the current Western lifestyle, in which activity levels and diet deviate substantially from the conditions under which our species’ metabolic physiology evolved [2]–[6]. Some propose that modern conveniences and mechanization lead to decreased physical activity and lower energy expenditure in industrialized societies [1]–[3]. Others hypothesize that changes in diet and energy intake are responsible, citing the relatively recent increase in energy dense foods, particularly processed foods high in fructose and other simple sugars that can depress energy expenditure and increase appetite and adiposity [4]–[7].

Determining which aspects of the Western lifestyle are truly aberrant for our species and pose the greatest risk of obesity is complicated by the conflicting and limited data on diet and metabolism in non-Western populations. For example, while Western diets are certainly more sugar-rich and energy-dense than more “traditional” diets and wild foods [4], [8], [9], many hunter-gatherers seasonally consume a large portion of their daily calories as honey [10], [11] (Fig. S2), which has high concentrations of glucose and fructose [12]. Similarly, while high activity levels have been reported in some subsistence farming populations [13]–[15], a recent meta-analysis of 98 diverse populations worldwide found no effect of socioeconomic development – a rough index of mechanization and diet – on daily energy expenditure or activity level [16]. Notably, metabolic measurements are lacking for hunter-gatherer societies, whose diet and lifestyle provide the best models for studies of human evolution [10].

In this study, we examined daily energy expenditure and physical activity level in Hadza foragers to test the hypothesis that hunter-gatherers expend more energy each day than subjects in market and farming economies. The Hadza are a population of hunter-gatherers living in a savannah-woodland environment in Northern Tanzania; their traditional foraging lifestyle has been documented extensively in previous work [17]. While no living population is a perfect model of our species’ past, the Hadza lifestyle is similar in critical ways to those of our Pleistocene ancestors. The Hadza hunt and gather on foot with bows, small axes, and digging sticks, without the aid of modern tools or equipment (e.g., no vehicles or guns). As in many other forager societies [10], there is a sexual division of foraging effort; Hadza men hunt game and gather honey, while Hadza women gather plant foods. Men’s forays are typically longer than women’s, as reflected in their mean daily travel distances (see below). Women typically forage in groups, while men tend to hunt alone [17]. As is typical among traditional-living Hadza, over 95% of their calories during this study came from wild foods, including tubers, berries, small- and large-game, baobab fruit, and honey [17] (Fig. S2).

We compared energy expenditure and body composition among the Hadza, measured using the doubly labeled water method [18], to similar data from other populations taken from previous studies [19]–[26] and new measurements of U.S. adults (Methods). Given their traditional, physically active lifestyle, we expected the Hadza to have lower body fat than individuals in Western populations. Further, if current models for obesity are correct, the Hadza, with their natural diet and lack of mechanization, should expend more energy than individuals living in market economies with comparatively sedentary lifestyles and highly-processed, sugar-rich diets.

We also measured daily walking distances (km/day) using wearable GPS devices, and the cost of walking (kCal kg^−1^ m^−1^) and resting metabolic rate (RMR, kCal kg^−1^ s^−1^) using a portable respirometry system (Text S1). Because it was not feasible to measure basal metabolic rate (BMR, kCal/day), we calculated physical activity level (PAL) as TEE/estimated BMR (Methods). Institutional approval and informed consent were obtained prior to data collection.

## Methods

### Subjects

We measured total daily energy expenditure (TEE, kCal/day) over an 11-day period in 30 Hadza adults (13 men ages 18–65, 17 women ages 18–75; Text S1). Ages, body weights, and other population statistics are given in Table 1.

**Table 1:** Population characteristics, energy expenditure, and body composition.

	HADZA		WESTERN		FARMING	
	Women	Men	Women	Men	Women	Men
N	17	13	186	53	14	11
Age (yr)	39.9±19.4	33.2±14.5	41.1±8.8	44.2±8.9	43.9±21.8	49.1±20.9
	*18–75*	*18–65*	*21–61*	*25–61*	*14–79*	*17–80*
Mass (kg)	43.4±6.4	50.9±5.4	74.4±12.8	81.0±11.1	48.1±6.9	54.7±2.9
	*34.0–55.0*	*42.5–58.2*	*49.5–117.7*	*57.2–101.3*	*39.0–62.8*	*49.5–58.7*
BMI (kg/m^2^)	20.2±1.7	20.3±1.3	27.5±4.5	25.6±2.7	20.7±3.2	21.2±1.6
	*17.0–23.9*	*19.1–23.4*	*19.5–39.4*	*19.5–30.0*	*17.2–28.9*	*19.5–24.0*
Body Fat %	20.9±4.6	13.5±4.2	37.9±7.0	22.5±5.0	27.3±5.3	16.0±3.3
	*12.4–27.7*	*7.4–23.1*	*11.9–53.3*	*10.2–32.9*	*18.8–36.9*	*9.8–21.1*
TEE (kCal/day)	1877±364	2649±395	2347±360	3053±464	2469±315	2855±435
	*1459–2596*	*2008–3363*	*1351–3978*	*2211–4682*	*1972–3202*	*2212–3374*
PAL (TEE/BMR)	1.78±0.30	2.26±0.48	1.68±0.22	1.81±0.21	2.11±0.30	2.08±0.26
	*1.44–2.53*	*1.67–2.96*	*1.21–2.54*	*1.56–2.42*	*1.44–2.63*	*1.65–2.51*

Values shown are means, ±standard deviations, and *ranges*. See Text S1 for details on comparative data sources. PAL for the Hadza was calculated using estimated BMR [27].

### Ethics Statement

Institutional approvals, including university (Washington University Institutional Review Board) and all cognizant local governmental agencies (including Tanzanian National Institute for Medical Research and Commission for Science and Technology), were obtained prior to conducting this study. All subjects gave their informed, verbal consent prior to participation. Verbal consent was deemed appropriate given the low literacy rates among traditional Hadza, and was specifically approved by university IRB and Tanzanian agencies. Each subject’s date and time of consent, and the researcher obtaining consent, were documented in the project field notes.

### Measuring TEE using Doubly Labeled Water

Total daily energy expenditure (TEE, kCal/day), was measured using the doubly labeled water (DLW) method, described in detail elsewhere [18]. Briefly, subjects were administered an oral dose of DLW (120 g; 10% H_2_^18^O, 6% ^2^H_2_O); dose containers were rinsed with bottled water three times to ensure the entire dose was consumed. Prior to dosing, and then at 12–24 hr, 4 d, 8 d, and 11 d after dose administration, urine samples were collected in dry, clean plastic cups, transferred to 2 ml cryovials (Sarstedt), frozen in liquid nitrogen in the field for 1–5 days, and then transferred to a −5°C freezer for long term storage. Urine collection days varied for some subjects due to logistical constraints. Urine samples were analyzed for ^18^O and ^2^H abundance at Baylor College of Medicine using gas isotope ratio mass spectrometry. The slope-intercept method was used to calculate dilution spaces and fat free mass (FFM); the rate of carbon dioxide production was calculated using a two-pool approach [18]. Carbon dioxide production was converted to TEE [18] using a respiratory quotient (RQ) of 0.85, following RQ values recorded during RMR measurements (Text S1).

Physical activity level (PAL) was calculated as TEE/estimated BMR for each subject following previous studies [13]–[16]. To estimate BMR for Hadza subjects, we entered each subject’s body mass and height into age-specific prediction equations developed in a large sample (n = 10,552) from a geographically broad set of populations that includes populations in sub-Saharan Africa [27].

### Resting Metabolic Rate and Walking Cost

Energy expenditures during resting and walking were measured using a portable, wearable respirometry system (Cosmed, K4b2) which measures both carbon dioxide production and oxygen consumption via “breath-by-breath” analysis. RMR was measured in 19 subjects (11 women, 8 men) while sitting quietly for 15–20 minutes (Text S1). Walking cost was measured in 14 subjects (5 women, 9 men) during over ground walking on a level trackway established near each camp on flat ground (Text S1). The minimum net cost of transport, COT_min_ (kCal kg^−1^ m^−1^), which for all but one subject occurred at the slowest walking speed, was averaged across subjects. Mean COT_min_ for the Hadza sample was compared to sample means measured in Western populations presented in a recent meta-analysis of walking cost [28].

To estimate daily walking cost (kCal/day) for each subject, each individual’s mean COT_min_ was multiplied by their body mass and daily travel distance. In order to measure daily travel distance, Hadza subjects wore a small global positioning system (GPS) device (Garmin 301 Forerunner) during daylight hours for their entire 11 day TEE measurement period. Occasionally, battery failure or other issues (e.g., accidentally switching the unit off) would prevent the device from capturing a full day of travel data. To ensure that incomplete measures of daily travel did not skew travel estimates downward, measurements were only considered to represent a full day of travel if the GPS device captured 10 or more hours that day; incomplete recordings were excluded from subsequent analysis.

### Comparative Data

We compared the Hadza to other populations using two sets of analyses, one that examined variation in TEE among *individuals*, and another that examined variation in mean TEE among *populations*. For analyses of TEE among individuals, data on TEE were gathered from previous DLW studies [16], [19]–[26] and from new measurements of TEE in U.S. adults (n = 68). For new measurements, TEE was assessed in free-living human subjects during 2-week periods using the DLW method [18]. The subjects were enrolled in a variety of studies involving diet and/or exercise interventions, but only pre-intervention data during weight stability are included in the current analysis. Additional TEE data was drawn from published values for individual subjects in Western (U.S. and Europe) countries [19]–[26]; here again, only pre-intervention or control group data were included in this analysis. Other comparative data was drawn from non-Western market economies [29], [30] and a subsistence agricultural population in the alteplano of Bolivia [13], [31]. Most subjects (n = 221) from comparative datasets had measured BMR data available as well, enabling us to calculate PAL as TEE/BMR. Individuals were grouped by lifestyle or economy for analyses: “hunter-gatherer” includes only Hadza subjects, “Western” includes individuals living in Europe or the U.S., “market” includes Westerners as well as other individuals living in non-Western, market economies (e.g., Siberia), and “farming” includes Bolivian farmers [13], [31].

For population-level analyses, we compared mean TEE for Hadza men and women to single-sex cohorts from a recent meta-analysis of TEE among a global sample of populations that included 198 single-sex cohorts representing 4,972 subjects [16]. Populations were classified as “hunter-gatherer” (i.e., Hadza), “market economy”, or “farming” based on descriptions of each population in the primary literature. The farming populations identified were located in Nigeria, Gambia, and Bolivia (note that only cohort means are available for Nigerian and Gambian farmers so these populations are not included in individual-level analyses). FFM was unavailable for most populations and thus total body mass was used as an index of body size. As a result, sex and age were significant predictors of TEE in these analyses (Table S1) because body fat percentage covaries with both.

### Statistical Analyses

TEE, body mass, and FFM were log_10_ transformed prior to analysis (JMP®); significance level for all analyses was p = 0.05. We tested for differences in TEE and PAL between lifestyle groups while controlling for FFM, age, and other variables using generalized linear modeling (GLM), an approach recommended by Tschop and colleagues [32]. Among the large Western sample (n = 239), a test for homogeneity of slopes revealed that men and women differed in the relationship between FFM and TEE (F(238) = 2.68, p<0.001). Slope heterogeneity violates the assumptions of ANCOVA and other GLM comparisons, and thus men and women were compared separately in multivariate analyses of TEE. Tests for homogeneity of slopes revealed that slopes were similar among Western and Hadza women (F(201) = 0.36, p = 0.55) and among Western and Hadza men (F(64) = 0.77, p = 0.38). Analyzing males and females separately does not affect the pattern of population comparisons; results from combined-sex analyses were similar (see below).

A similar approach was used to compare population means. A test for homogeneity of slopes revealed similar slopes between TEE and body mass in male and female cohorts (F(162) = 0.10, p = 0.75). TEE in males was significantly greater than in female populations after controlling for body mass (F(162) = 86.75, p<0.001, ANCOVA), most likely due to the greater mean body fat percentage in females.

## Results

Hadza were highly active and lean, with body fat percentages on the low end of the normal healthy range for Western populations [33] (Table 1). TEE among Hadza adults was strongly related to body size, specifically fat free mass (FFM) (r^2^ = 0.66, n = 30, p<0.001; Table S1). In multivariate comparisons controlling for mass, height, sex, and age, body fat percentages for Hadza adults were lower than individuals from Western (U.S. and Europe) populations (F(228) = 22.72, p<0.001). Body fat percentages, TEE, and other population characteristics are listed in Table 1.

**Figure 1:**
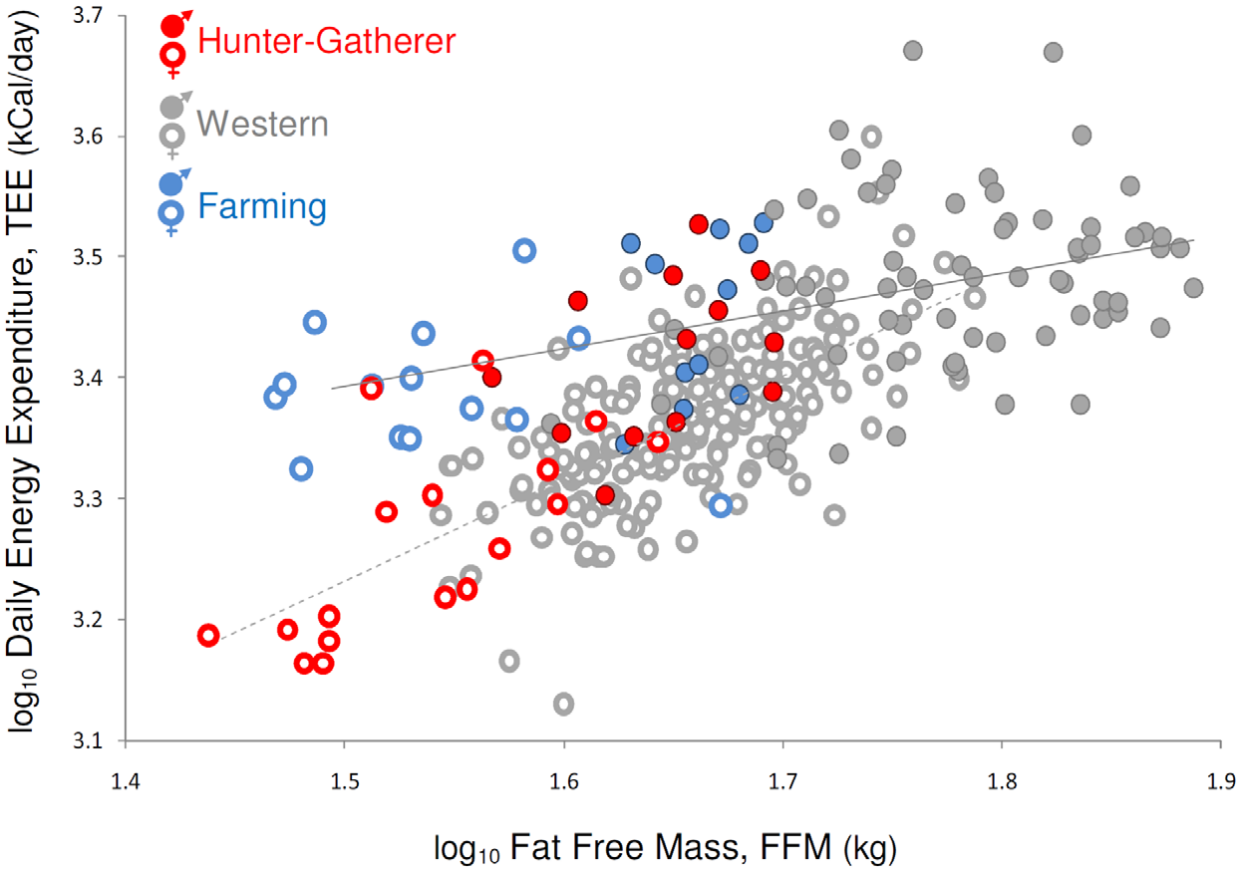
Individual comparisons of TEE and FFM. Energy expenditure for Hadza hunter-gatherers (red circles) was similar to that of Westerners (gray [19]–[26]). Bolivian women farmers (blue open circles [13], [31]) had higher TEE than either Hadza or Western women. Trendlines are ordinary least squares regressions through Western men (solid line) and Western women (dashed line).

Contrary to expectations, measures of TEE among Hadza adults were similar to those in Western (U.S. and Europe) populations. In multivariate comparisons of TEE controlling for FFM and age, Hadza women’s energy expenditure was similar to that of Western women (n = 186) and Hadza men’s TEE was similar to Western men (n = 53); lifestyle had no effect on TEE (women: F(139) = 0.18, p = 0.67; men: F(49) = 0.17, p = 0.68) (Fig. 1, Table S1). Results were unchanged when Hadza were compared to all market-economy individuals, or when body mass was substituted for FFM (Table S1), or when sexes were combined for analyses (lifestyle: F(189) = 0.25, p = 0.62). Including fat mass as an independent variable modestly improved the fit of multivariate models for TEE but did not affect the pattern of results (Table S1). The absence of significant differences does not appear to result from small sample sizes for the Hadza. Power analyses indicated that sample sizes were sufficient to detect a 4.2% difference in mean TEE (Hadza vs. Western, α = 0.05) in comparisons among women (power 97%) and 7.6% difference among men (power 93%).

**Figure 2:**
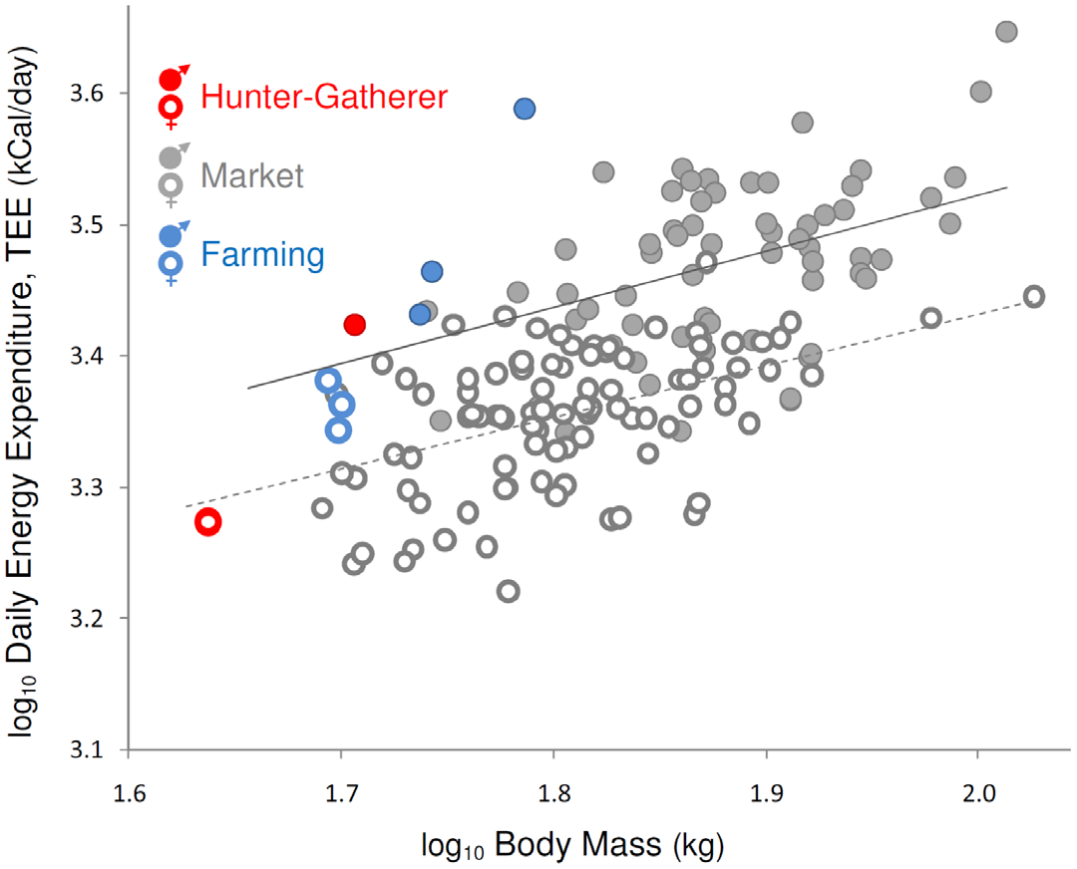
Population comparisons of TEE. Energy expenditure among Hadza hunter-gatherers (red circles) was similar to populations in market economies; subsistence farming populations (Nigeria, Gambia, Bolivia; blue circles) had higher TEE than other groups. All non-Hadza data from [16] (Text S1). Each symbol is a single-sex population mean; male and female means are plotted separately for mixed-sex studies. Ordinary least squares regression lines are shown for all men (filled circles, solid line) and all women (open circles, dashed line). When controlling for body mass, men had higher TEE than women (F(162) = 86.75, p<0.001).

Similarity in TEE between Hadza and other populations was also evident when population means were compared. In a multivariate analysis controlling for sex, age, and body mass, TEE among Hadza hunter-gatherers did not differ (t(155) = −0.35, p = 0.73) from populations in market economies (Fig. 2, Table S1). Only farming populations had greater TEE than predicted for their body size. In comparisons among individual subjects, female Bolivian farmers [13] had higher TEE than Western and Hadza women (p<0.001 both comparisons, Table 1), and farming groups (n = 3) had consistently greater TEE in population comparisons (t = 2.76, p = 0.006, Table S1) (Fig. 1, 2).

Estimated physical activities levels (PAL, calculated as TEE/estimated BMR), suggest that Hadza adults spend a smaller portion of TEE on BMR than Westerners. Hadza men had an estimated PAL of 2.26±0.48, significantly greater than observed PAL in Western men (n = 31, PAL = 1.81±0.21) (F(43) = 13.07, p = 0.001), while estimated PAL for Hadza women (1.78±0.30) was slightly higher than that of Western women (n = 145, PAL = 1.68±0.22) (F(162) = 3.80, p = 0.05) when controlling for age (Table S1). Regressing TEE on estimated BMR suggests that group differences in PAL were related to differences in body size, as the Hadza are significantly smaller than their Western counterparts (Table 1). In a multivariate analysis controlling for age and sex, the relationship between TEE and estimated BMR did not differ between Hadza and Western subjects (F(239) = 0.73, p = 0.39) (Fig. S3). However, because TEE is correlated with estimated BMR with a slope <1.0, PAL (the ratio of TEE/BMR) tends to be greater among smaller individuals; this is particularly evident among men in our sample (Fig. S3).

Daily walking distances for Hadza women (mean 5.8, std. dev. ±1.7 km/day) and men (11.4±2.1 km/day) were significantly different (p<0.001, t-test), consistent with previous measurements in hunting and gathering societies [10]. However, individual variation in daily walking distance did not explain variation in TEE. Estimated daily energy expenditure on walking (kCal/day) accounted for an average of 6.7% (±1.9%) of TEE among Hadza women and 11.0% (±3.4%) among Hadza men (Text S1), but TEE was not correlated with mean daily travel distance (F(28) = 0.75, p = 0.39) (Table S1). Similarly, TEE of Hadza women who were pregnant or lactating (n = 8; 1 pregnant, 7 lactating) was no different from other Hadza women (n = 9; F(16) = 0.96, p = 0.35) after controlling for FFM (Table S1).

While the Hadza likely perform traditional foraging tasks (e.g., digging tubers or chopping tree limbs for honey) more efficiently than unacculturated Westerners can [34], comparisons of activities common across cultures do not indicate that Hadza muscle and locomotor physiology inherently more efficient. The energy cost of walking (kCal kg^−1^ m^−1^) for Hadza adults was well within the range of values reported for Western subjects: of 20 U.S and European populations included in a recent meta-analysis of treadmill walking cost [28], 14 had mean COT_min_ values below the Hadza mean (Supporting Information, Fig. S1). RMR for Hadza adults measured while sitting averaged 11% above predicted BMR [27], within the range of values (7–35%) reported for other populations [35].

## Discussion

**Figure 3:**
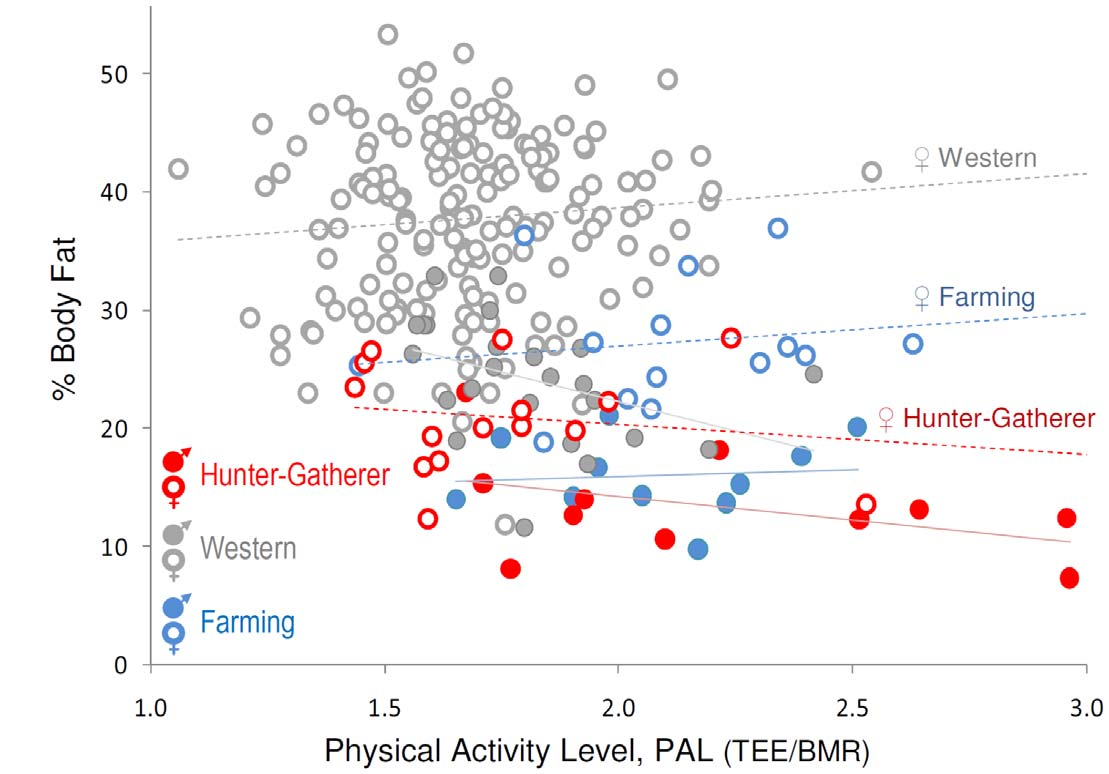
Percent body fat plotted against physical activity level, PAL. Hadza BMRs are estimated (Methods). Trendlines shown separately for each sex/lifestyle group; dashed lines indicate female groups. Group body fat differences are significant (p<0.001), but slopes for %body fat versus PAL are not (Table S1).

Measurements of TEE among Hadza hunter-gatherers challenge the view that Western lifestyles result in abnormally low energy expenditure, and that decreased energy expenditure is a primary cause of obesity in developed countries. Despite high PAL and dependence on wild foods, Hadza TEE was similar to Westerners and others in market economies (Fig. 1, 2). Further, while Hadza differed from Western populations in body fat percentage (F(202) = 44.05, p<0.001), variation in adiposity both within and between populations was not correlated with PAL (F(207) = 0.36, p = 0.55) (Fig. 3) nor with TEE (F(209) = 3.02, p = 0.08, β = 12.06; note that the effect of TEE on adiposity, while not statistically significant, is positive in this sample). The lack of correspondence between TEE, PAL, and adiposity in our Hadza and comparative samples is consistent with previous DLW studies in Western populations [36]–[38]. The similarity in TEE among Hadza hunter-gatherers and Westerners suggests that even dramatic differences in lifestyle may have a negligible effect on TEE, and is consistent with the view [4]–[7], [16] that differences in obesity prevalence between populations result primarily from differences in energy intake rather than expenditure. TEE and PAL measurements of other traditional populations, preferably hunter-gatherers, are needed to assess whether the pattern of energy expenditure among the Hadza is typical of human foragers.

It is important to note that this was not an intervention study; we examined habitual TEE, PAL, and body composition in hunter-gatherers and Westerners, but did not examine the effects of imposing increased physical activity on Westerners. Physical activity has important, positive effects on health [39], and increased physical activity has been shown to play an important role in weight loss and weight-maintenance programs [40]. Some studies of self-reported activity level have even suggested that habitual activity may help prevent unhealthy weight gain, although the evidence is mixed [40]. More work is needed to integrate results from intervention studies of PAL and TEE with population-level comparisons of habitual energy expenditure.

Our results indicate that active, “traditional” lifestyles may not protect against obesity if diets change to promote increased caloric consumption. Thus, efforts to supplement diets of healthy populations in developing regions must avoid inundating these individuals with highly-processed, energy-dense but nutrient-poor foods. Since energy throughput in these populations is unlikely to burn the extra calories provided, such efforts may unintentionally increase the incidence of excess adiposity and associated metabolic complications such as insulin resistance. Indeed, processed, energy-dense foods have been linked to insulin resistance and cardiovascular disease among Australian foragers transitioning to village life [41].

The similarity in TEE between the Hadza and Western populations is counterintuitive given the Hadza’s physically active lifestyle and elevated PAL. TEE among Hadza and Westerners was indistinguishable when controlling for lean mass and fat mass (common proxies for non-activity metabolic costs) despite differences in lifestyle and estimated PAL. These results, and the possible interaction between PAL and body size (Fig. S3), indicate more work on the physiology of traditional populations is clearly needed. Moreover, the lack of correspondence between TEE and daily walking distance (a large component of Hadza daily activity), or between TEE and maternal status (pregnant/nursing or not), along with other investigations of forager physiology, suggest that interactions between metabolic physiology, physical activity, and the environment are more complex than often thought. For example, work with Ache foragers in Paraguay has shown that levels of leptin, critical in fat sequestration, and testosterone, an anabolic hormone, are substantially lower than levels seen in U.S. adults [42], [43]. And studies of pregnant and lactating women in traditional cultures have shown that changes in both behavior (e.g., work load) and physiology (e.g., BMR) enable them to maintain TEE at levels similar to those of their Western counterparts [44], [45]. Studies such as these, as well as results here, suggest that physical activity may be only one piece of a dynamic metabolic strategy that is continuously responding to changes in energy availability and demand. Recent work examining the body’s complex physiological responses to dieting and weight loss [46] supports this view.

Data on hunter-gatherer TEE provide additional perspectives on Paleolithic humans and on the origins of farming. While the lifestyle of late Pleistocene hunter-gatherers was no doubt highly active as seen in foragers today, our results suggest that their daily energy requirements were likely no different than current Western populations. And rather than decreasing the work needed to find food, early agriculture may reflect an effort to improve food security and predictability, even at the cost of slightly higher energy demands. The greater energy demands of traditional farming lifestyles evident in this study (Fig. 1, 2) suggest that the adoption of agriculture brought with it an increased workload for Neolithic foragers. This view is consistent with Sahlins’ [47] proposition that Pleistocene hunter-gatherers enjoyed an “original affluence,” spending only a moderate amount of time on subsistence work each day, as well as a recent study indicating that Neolithic foragers were no less productive than early farmers in obtaining food [48].

Like other complex, continuous traits (e.g., stature), environment can clearly influence TEE, as is evident in the elevated energy expenditures of traditional farmers (Table 1). Nonetheless, TEE is remarkably similar across a broad, global sample of populations that span a range of economies, climates, and lifestyles (Fig. 1, 2). Not only is TEE statistically indistinguishable between Westerners and Hadza foragers, but the range of TEE within Western, foraging, and farming populations largely overlap, both at the individual and population levels (Table 1, Fig. 1, 2). We hypothesize that TEE may be a relatively stable, constrained physiological trait for the human species, more a product of our common genetic inheritance than our diverse lifestyles. A growing body of work on mammalian metabolism is revealing that species’ metabolic rates reflect their evolutionary history, as TEE responds over evolutionary time to ecological pressures such as food availability and predation risk [49], [50]. In this light, it is interesting to consider human TEE as an evolved trait shaped by natural selection. Humans are known to have greater TEE than orangutans [50], a closely related ape, but have low TEE compared to other eutherian mammals [50], [51]. Data from other primate species are needed to fit the human metabolic strategy into a comprehensive evolutionary context.

## Supporting Information

Figure S1 **Walking cost in Hadza adults compared to other populations.** Mean COT_min_ values for the Hadza (n = 14) are within the range of twenty Western populations reported in a recent meta-analysis [28]. Error bars indicate standard deviations. Note that the Hadza bar represents a mean of individual subjects, while the Western bar represents the mean of 20 population means [28].
(TIF)

Figure S2 **Key foods in the Hadza diet during this study as a percentage of total calories brought back to camp.**
(TIF)

Figure S3 **The effect of body size on PAL in the current dataset. A**. TEE versus estimated BMR for Hadza and Western adults. Symbols as in Figure 1. **B**. PAL versus body mass.
(TIF)

Table S1 **Results of multivariate analyses.**
(PDF)

Text S1 **Describes additional details of the methods used to collect and analyze data, as well as additional information regarding the Hadza population.**
(PDF)

## References

[1] World Health Organization (2011). Obesity and Overweight.. http://www.who.int/mediacentre/factsheets/fs311/en/index.html.

[2] Popkin BM (2005). Using research on the obesity pandemic as a guide to a unified vision of nutrition.. Public Health Nutr.

[3] Prentice AM, Jebb SA (1995). Obesity in Britain: gluttony or sloth?. BMJ.

[4] Prentice AM, Jebb SA (2003). Fast foods, energy density and obesity: a possible mechanistic link Obesity Reviews.

[5] Isganaitis E, Lustig RH (2005). Fast food, central nervous system insulin resistance, and obesity.. Arterioscler Thromb Vasc Biol.

[6] Stanhope KL, Havel PJ (2008). Endocrine and metabolic effects of consuming beverages sweetened with fructose, glucose, sucrose, or high-fructose corn syrup.. Am J Clin Nutr.

[7] Swinburn BA, Sacks G, Hall KD, McPherson K, Finegood DT (2011). The global obesity pandemic: shaped by global drivers and local environments.. Lancet.

[8] Schoeninger MJ, Murray S, Bunn HT, Marlett JA (2001). Composition of tubers used by Hadza foragers of Tanzania.. J Food Comp Analysis.

[9] Murray S, Schoeninger MJ, Bunn HT, Pickering TR, Marlett JA (2001). Nutritional composition of some wild plant foods and honey used by Hadza foragers of Tanzania.. J Food Comp Analysis.

[10] Marlowe FW (2005). Hunter-gatherers and human evolution.. Evol Anth.

[11] Marlowe FW, Berbesque JC (2009). Tubers as fallback foods and their impact on Hadza hunter-gatherers.. Am J Phys Anth.

[12] Ischayek JI, Kern M (2006). US honeys varying in glucose and fructose content elicit similar glycemic indexes.. J Am Diet Assoc.

[13] Kashiwazaki H, Dejima Y, Orias-Rivera J, Coward WA (1995). Energy expenditure determined by the doubly labeled water method in Bolivian Aymara living in a high altitude agropastoral community.. Am J Clin Nutr.

[14] Esparza J, Fox C, Harper IT, Bennett PH, Schulz LO (2000). Daily energy expenditure in Mexican and USA Pima indians: low physical activity as a possible cause of obesity.. Int J Obes Relat Metab Disord.

[15] Dufour DL, Piperata BA (2008). Energy expenditure among farmers in developing countries: what do we know?. Am J Hum Biol.

[16] Dugas LR, Harders R, Merrill S, Ebersole K, Shoham DA (2011). Energy expenditure in adults living in developing compared with industrialized countries: a meta-analysis of doubly labeled water studies.. Am J Clin Nutr.

[17] Marlowe FW (2010). The Hadza: Hunter-Gatherers of Tanzania. Univ. California Berkeley.. 336 p.

[18] “Assessment of Body Composition, Total Energy Expenditure in Humans Using Stable Isotope Techniques” (2009). IAEA Human Health Series 3.

[19] Davidson L, McNeill G, Haggarty P, Smith JS, Franklin MF (1997). Freeliving energy expenditure of adult men assessed by continuous heart-rate monitoring and doubly-labelled water.. Br J Nutr.

[20] Prentice AM, Black AE, Coward WA, Davies HL, Goldberg GR (1986). High levels of energy expenditure in obese women.. Br Med J (Clin Res Ed).

[21] Racette SB, Schoeller DA, Kushner RF, Neil KM, Herling-Iaffaldano K (1995). Effects of aerobic exercise and dietary carbohydrate on energy expenditure and body composition during weight reduction in obese women.. Am J Clin Nutr.

[22] Racette SB, Schoeller DA, Kushner RF, Neil KM (1995). Exercise enhances dietary compliance during moderate energy restriction in obese women.. Am J Clin Nutr.

[23] Racette SB, Weiss EP, Villareal DT, Arif H, Steger-May K (2006). One year of caloric restriction in humans: feasibility and effects on body composition and abdominal adipose tissue.. J Gerontol A Biol Sci Med Sci.

[24] Schulz S, Westerterp KR, Bruck K (1989). Comparison of energy expenditure by the doubly labeled water technique with energy intake, heart rate, and activity recording in man.. Am J Clin Nutr 491146–1154.

[25] Seale JL, Rumpler WV, Conway JM, Miles CW (1990). Comparison of doubly labeled water, intake-balance, and direct- and indirectcalorimetry methods for measuring energy expenditure in adult men.. Am J Clin Nutr.

[26] Welle S, Forbes GB, Statt M, Barnard RR, Amatruda JM (1992). Energy expenditure under free-living conditions in normal-weight and overweight women.. Am J Clin Nutr.

[27] Henry CJ (2005). Basal metabolic rate studies in humans: measurement and development of new equations.. Public Health Nutr.

[28] Rubenson J, Heliams DB, Maloney SK, Withers PC, Lloyd DG (2007). Reappraisal of the comparative cost of human locomotion using gait-specific allometric analyses.. J Exp Biol.

[29] Snodgrass JJ, Leonard WR, Tarskaia LA, Schoeller DA (2006). Total energy expenditure in the Yakut (Sakha) of Siberia as measured by the doubly labeled water method.. Am J Clin Nutr.

[30] Stein TP, Johnston FE, Greiner L (1988). Energy expenditure and socioeconomic status in Guatemala as measured by the doubly labelled water method.. Am J Clin Nutr.

[31] Kashiwazaki H, Uenishi K, Kobayashi T, Rivera JO, Coward WA (2009). Year-round high physical activity levels in agropastoralists of Bolivian Andes: results from repeated measurements of DLW method in peak and slack seasons of agricultural activities. Am J Hum Biol..

[32] Tschöp MH, Speakman JR, Arch JR, Auwerx J, Brüning JC (2011). A guide to analysis of mouse energy metabolism.. Nat Methods.

[33] Gallagher D, Heymsfield SB, Heo M, Jebb SA, Murgatroyd PR (2000). Healthy percentage body fat ranges: an approach for developing guidelines based on body mass index.. Am J Clin Nutr.

[34] Kaplan HS, Hill KR, Lancaster JB, Hurtado AM (2000). A theory of human life history evolution: diet, intelligence, and longevity.. Evol Anth.

[35] Kanade AN, Gokhale MK, Rao S (2001). Energy costs of standard activities among Indian adults.. Eur J Clin Nutr.

[36] Speakman JR, Westerterp KR (2010). Associations between energy demands, physical activity, and body composition in adult humans between 18 and 96 y of age. Am J Clin Nutr..

[37] Goran MI, Hunter G, Nagy TR, Johnson R (1997). Physical activity related energy expenditure and fat mass in young children. Int J Obes Relat Metab Disord..

[38] Westerterp KR (2010). Physical activity, food intake, and body weight regulation: insights from doubly labeled water studies. Nutr Rev..

[39] World Health Organization (2010). Global recommendations on physical activity for health. World Health Organization, Geneva.. 60 p.

[40] Chaput JP, Klingenberg L, Rosenkilde M, Gilbert JA, Tremblay A (2011). Physical activity plays an important role in body weight regulation. J Obes. 2011.. pii.

[41] O’Dea K (1991). Westernization and non-insulin-dependent diabetes in Australian Aborigines.. Ethn Dis.

[42] Bribiescas RG (2001). Serum leptin levels and anthropometric correlates in Ache Amerindians of eastern Paraguay.. Am J Phys Anth.

[43] Ellison PT, Bribiescas RG, Bentley GR, Campbell BC, Lipson SF (2002). Population variation in age-related decline in male salivary testosterone.. Hum Reprod.

[44] Butte NF, King JC (2005). Energy requirements during pregnancy and lactation.. Public Health Nutr.

[45] Dufour DL, Sauther ML (2002). Comparative and evolutionary dimensions of the energetics of human pregnancy and lactation.. Am J Hum Biol.

[46] Sumithran P, Prendergast LA, Delbridge E, Purcell K, Shulkes A (2011). Long-term persistence of hormonal adaptations to weight loss.. New Eng J Med.

[47] Sahlins M (1972). Stone Age Economics. Aldine, Chicago.. 348 p.

[48] Bowles S (2011). Cultivation of cereals by the first farmers was not more productive than foraging.. Proc Natl Acad Sci USA.

[49] Pontzer H, Kamilar JM (2009). Great ranging associated with greater reproductive investment in mammals.. Proc Natl Acad Sci USA.

[50] Pontzer H, Raichlen DA, Shumaker RW, Ocobock C, Wich SA (2010). Metabolic adaptation for low energy throughput in orangutans.. Proc Natl Acad Sci USA.

[51] Hayes M, Chustek M, Heshka S, Wang Z, Pietrobelli A (2005). Low physical activity levels of modern *Homo sapiens* among free-ranging mammals.. Int J Obes.

