# Supplementary figures and images for: Hunter-Gatherer Energetics and Human Obesity
Source: PLoS One. 2012 Jul 25;7(7):e40503. doi: 10.1371/journal.pone.0040503 (PMC3405064; doi:10.1371/journal.pone.0040503)

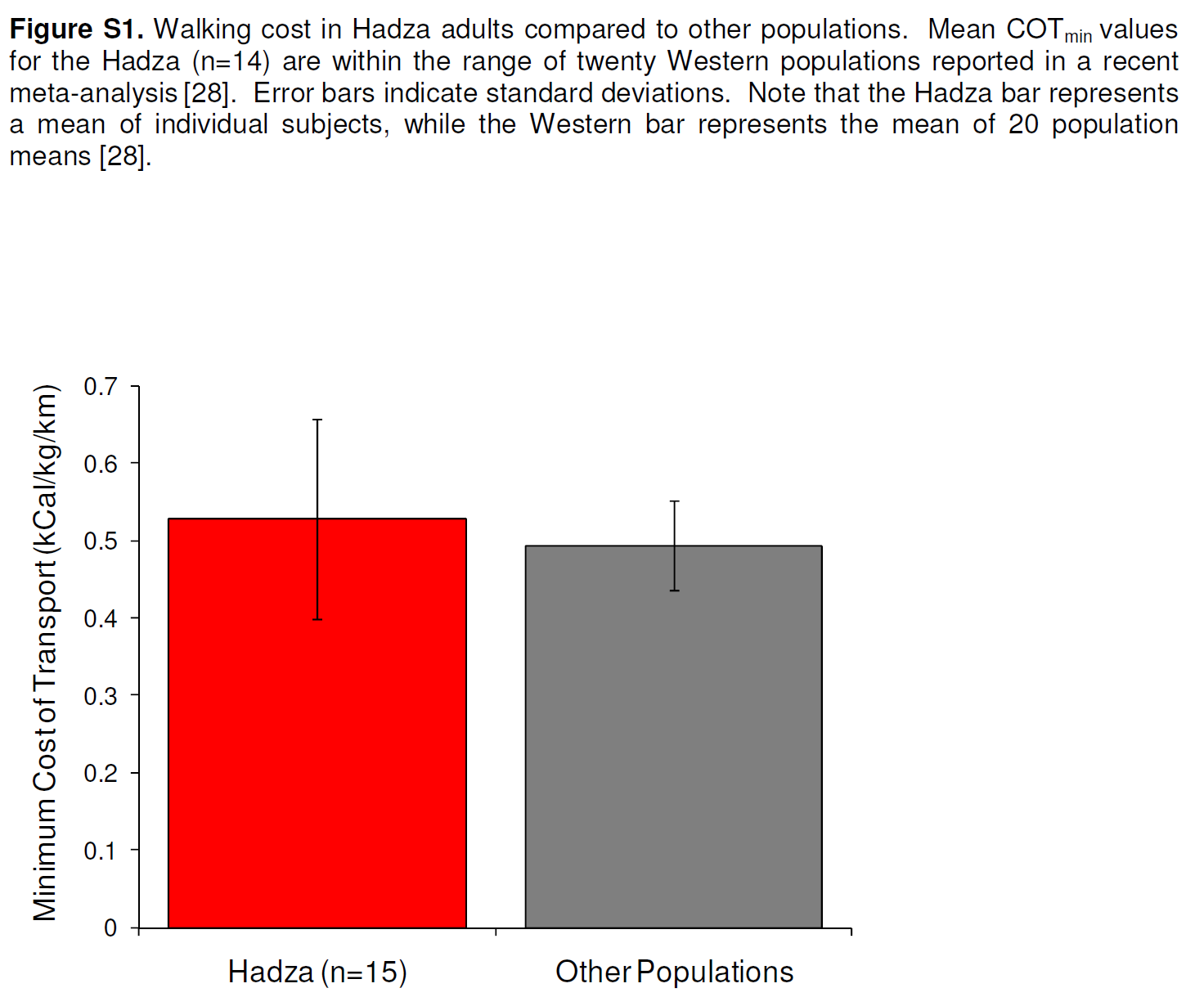

Supplement: Figure S1 — Walking cost in Hadza adults compared to other populations. Mean COTmin values for the Hadza (n = 14) are within the range of twenty Western populations reported in a recent meta-analysis [28]. Error bars indicate standard deviations. Note that the Hadza bar represents a mean of individual subjects, while the Western bar represents the mean of 20 population means [28]. (TIF) [file pone.0040503.s001.tif]

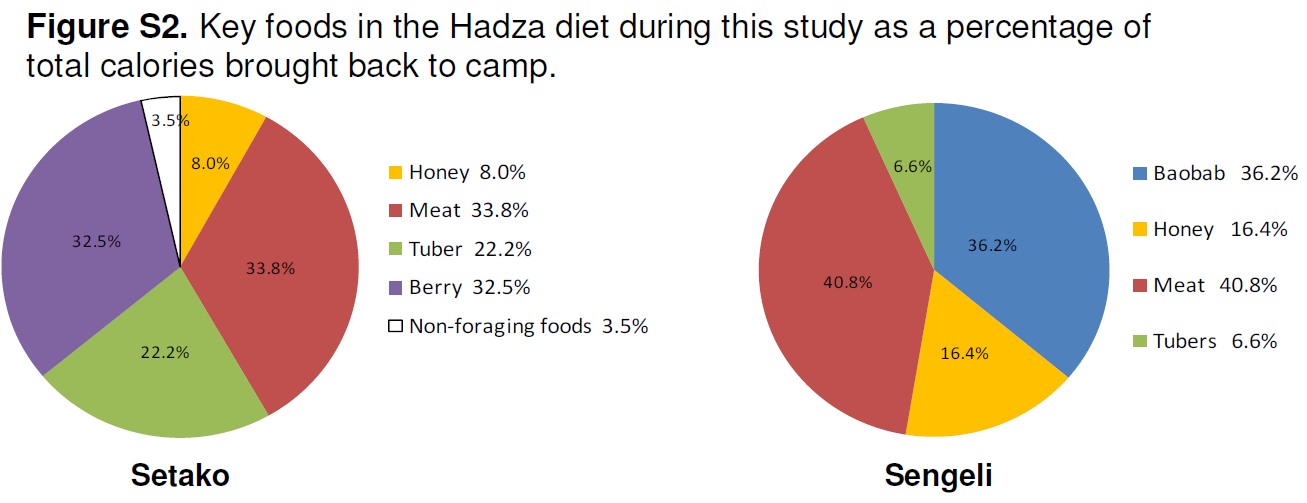

Supplement: Figure S2 — Key foods in the Hadza diet during this study as a percentage of total calories brought back to camp. (TIF) [file pone.0040503.s002.tif]

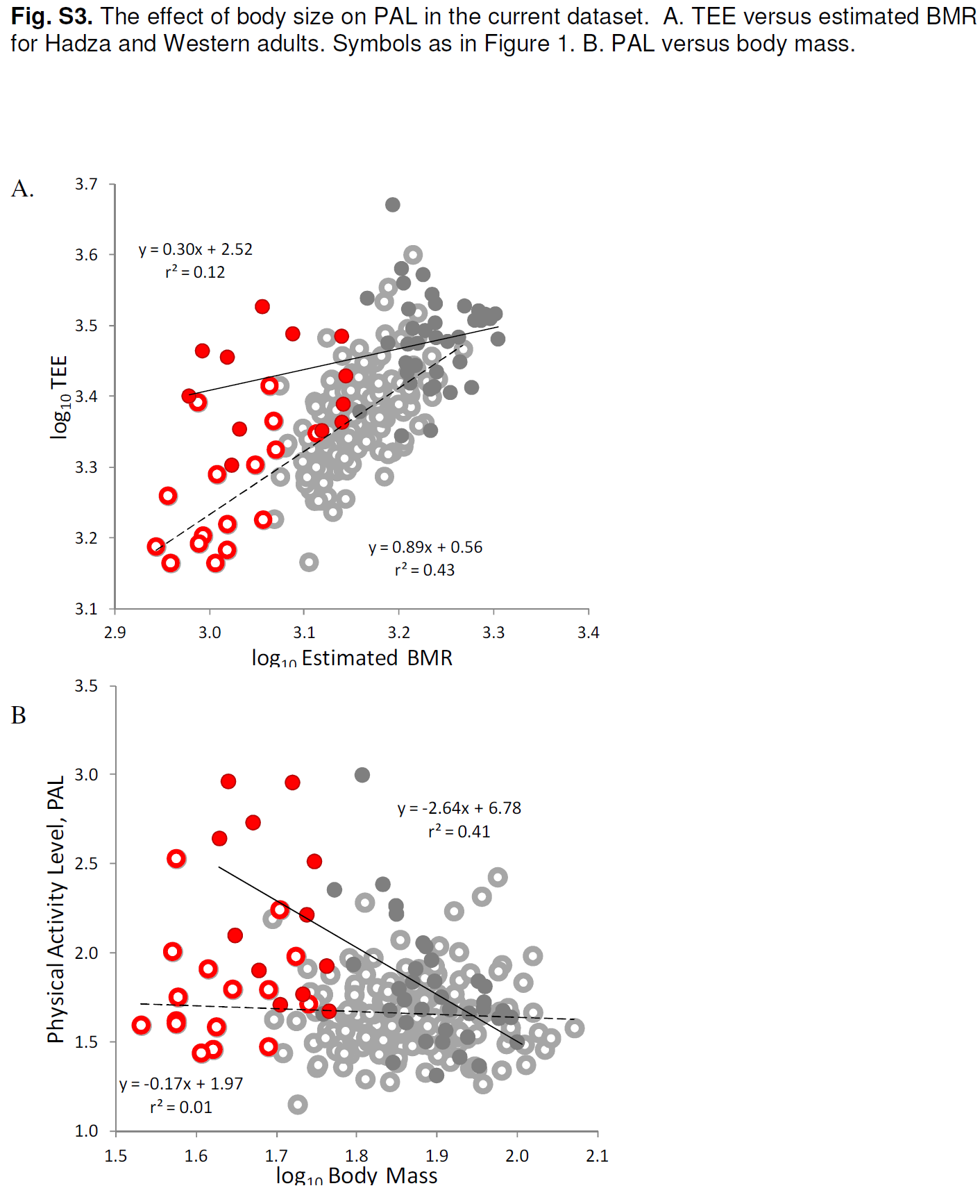

Supplement: Figure S3 — The effect of body size on PAL in the current dataset. A. TEE versus estimated BMR for Hadza and Western adults. Symbols as in Figure 1. B. PAL versus body mass. (TIF) [file pone.0040503.s003.tif]
