# Supplementary material for: Hunter-Gatherer Energetics and Human Obesity
Source: PLoS One. 2012 Jul 25;7(7):e40503. doi: 10.1371/journal.pone.0040503 (PMC3405064; doi:10.1371/journal.pone.0040503)
Supplement: Table S1 — Results of multivariate analyses. (PDF) [file pone.0040503.s004.pdf]

Table S1. Model summaries for statistical comparisons between Hadza hunter-gatherers, subjects in market economies, and farmers. **A.** Individual subject comparisons. **B.** Population mean comparisons.

A. Individual Subjects

Hadza Adults

Response	*df*	*Model r^2*	*Predictor*	*F-ratio*	*p*
TEE	29	0.69	FFM	11.90	<0.001
			Age	0.18	0.67
			Sex	2.36	0.14

Response	*df*	*Model r^2*	*Predictor*	*F-ratio*	*p*
TEE	29	0.64	Body Mass	6.17	0.02
			Age	0.39	0.54
			Sex	12.98	0.001

Response	*df*	*Model r^2*	*Predictor*	*F-ratio*	*p*
TEE	29	0.65	FFM	16.35	<0.001
			Daily Travel	0.77	0.44

Response	*df*	*Model r^2*	*Predictor*	*F-ratio*	*p*
TEE	16	0.50	FFM	13.24	0.003
			Pregnant/Lactating *versus* Not	0.91	0.35

Women: Hadza vs Western

Response	*df*	*Model r^2*	*Predictor*	*F-ratio*	*p*
TEE	139	0.54	FFM	100.05	<0.001
			Age	0.67	0.01
			Lifestyle	0.18	0.67

Response	*df*	*Model r^2*	*Predictor*	*F-ratio*	*p*
PAL	162	0.06	Age	5.82	0.02
			Lifestyle	3.80	0.05

Men: Hadza vs Western

Response	*df*	*Model r^2*	*Predictor*	*F-ratio*	*p*
TEE	49	0.25	FFM	5.41	0.02
			Age	0.86	0.36
			Lifestyle	0.17	0.68

Response	*df*	*Model r^2*	*Predictor*	*F-ratio*	*p*
PAL	43	0.26	Age	1.54	0.22
			Lifestyle	8.17	0.01

Women: Hadza vs. Market Economy (includes Western and ref. 29,30)

Response	*df*	*Model r^2*	*Predictor*	*F-ratio*	*p*
TEE	168	0.50	FFM	115.82	<0.001
			Age	2.32	0.13
			Lifestyle	1.05	0.31

Response	*df*	*Model r^2*	*Predictor*	*F-ratio*	*p*
TEE	193	0.48	Body Mass	122.40	<0.001
			Age	0.01	0.93
			Lifestyle	0.38	0.54

Response	*df*	*Model r^2*	*Predictor*	*F-ratio*	*p*
TEE	168	0.53	FFM	64.86	<0.001
			Fat Mass	8.03	0.01
			Age	0.92	0.34
			Lifestyle	0.09	0.76

Men: Hadza vs. Market Economy (includes Western and ref. 29,30)

Response	*df*	*Model r^2*	*Predictor*	*F-ratio*	*p*
TEE	63	0.26	FFM	12.16	<0.001
			Age	1.47	0.23
			Lifestyle	0.01	0.93

Response	*df*	*Model r^2*	*Predictor*	*F-ratio*	*p*
TEE	63	0.17	Body Mass	4.00	0.05
			Age	0.10	0.76
			Lifestyle	0.15	0.70

Response	*df*	*Model r^2*	*Predictor*	*F-ratio*	*p*
TEE	63	0.32	FFM	16.81	<0.001
			Fat Mass	4.67	0.04
			Age	2.52	0.12
			Lifestyle	1.46	0.23

Body Fat %: Hadza, Western, and Farming Populations

Response	*df*	*Model r^2*	*Predictor*	*F-ratio*	*p*
BodyFat%	207	0.59	Age	3.90	0.05
			Sex	99.54	<0.001
			Lifestyle	45.96	<0.001
			PAL	0.36	0.55

Response	*df*	*Model r^2*	*Predictor*	*F-ratio*	*p*
BodyFat%	209	0.60	Age	3.09	0.08
			Sex	106.31	<0.001
			Lifestyle	47.54	<0.001
			PAL	3.02	0.08, β=12.06

B. Population Means

Response	*df*	*Model r^2*	*Predictor*	*F-ratio*	*p*
TEE	155	0.79	Body Mass	78.91	<0.001
			Sex	143.70	<0.001
			Age	112.00	<0.001
			Lifestyle	3.32	0.02
			Farming		*0.01*
			Hunt./Gath.		*0.73*
			Market		*0.17*
