# Supplementary material for: Hunter-Gatherer Energetics and Human Obesity
Source: PLoS One. 2012 Jul 25;7(7):e40503. doi: 10.1371/journal.pone.0040503 (PMC3405064; doi:10.1371/journal.pone.0040503)
Supplement: Text S1 — Describes additional details of the methods used to collect and analyze data, as well as additional information regarding the Hadza population. (PDF) [file pone.0040503.s005.pdf]

Supporting Information

Pontzer et al. *Hunter Gatherer Energetics and the Origin of Human Obesity*

Hadza TEE Measurements

Total daily energy expenditure (TEE; kCal/day) was assessed using the doubly labeled water (DLW) method as outlined in the text and described in detail elsewhere [18]. The DLW method measures the amount of carbon dioxide produced and converts this rate of carbon dioxide production to a rate of energy expenditure using a respiratory quotient (RQ) of oxygen consumed:carbon dioxide produced. Following previous human studies [18], we used a RQ of 0.85. While RQ can be affected by differences in diet, an RQ=0.85 was statistically indistinguishable from the mean RQ measured during RMR trials (see below) for 21 Hadza subjects (mean=0.88 ±0.12), indicating that the RQ=0.85 value typically used in human DLW studies was accurate for calculating TEE for the Hadza sample. In fact, using the slightly (but not significantly) higher respiratory quotient measured among the Hadza would result in lowering their calculated TEE by approximately 2-3%. However, we use the more common value of RQ=0.85 here because it is more conservative for our comparisons of TEE.

TEE was measured at two different camps: Setako (n=18) and Sengeli (n=12). We measured TEE in Setako camp in September of 2009 and May-June of 2010, and in Sengeli camp in June 2010. Both September and May-June fall within "dry" seasons in this region [10]. Weather throughout the measurement periods in both camps was sunny and dry, with occasional brief rain showers in the evenings; there were no weather events (e.g., long periods of rain) that affected normal foraging or camp activity. The Hadza do not adhere to a daily or weekly calendar, and thus weekly variation in energy expenditure that might be expected in Western populations is not a likely source of variation in TEE among the Hadza. Two individuals were measured at Setako in both 2009 and 2010 sessions. TEE measurements for these individuals

differed by 9% between sessions. Similarly, one subject was measured at both Setako and Sengeli in 2010; the difference in his TEE measurements was 11%. This degree of variation likely results primarily from fluctuation in energy expenditure, as the error variance inherent in the DLW method is approximately 3-5% [18]. For these three subjects, measurements taken during different sessions were averaged, and the mean values used for subsequent analysis.

TEE was positively correlated with body mass among Hadza adults (r^2=0.45, p<0.001, df=29). However, as in other populations, fat free mass (FFM, kg), was a better predictor of TEE, explaining 66% of the variance in TEE in a pooled sample of Hadza adults (r^2=0.66, n=30, p<0.001). Further, FFM was the only significant predictor of TEE (F(29)=11.90, p=0.002) in a multivariate analysis that included age (F(29)=0.18, p=0.67) and sex (F(29)=2.36, p=0.14). Body mass index (BMI, kg/m^2) for Hadza men (20.3 ±0.4) and women (20.2 ±0.4) were similar (p=0.93 Student's t-test), while body fat percentage was greater in women (21% ±1.2%) than men (13% ±1.1%; p<0.001). Mean BMI and body fat percentage were on the low end of the normal range for Western populations [31] (Table 1).

Hadza RMR, estimated BMR, and Walking Cost

During RMR measurements subjects sat in a chair or on the ground (whichever they preferred) and rested quietly for 15 to 20 minutes. RMR measurements were taken in the early mornings or early evenings. We were not able to control whether subjects had eaten prior to RMR measurements. Energy expenditure during the last 10 minutes of data collection was averaged to determine RMR. RMR measures trended slightly higher (mean +11% for pooled subjects, p=0.06 for paired t-test) than estimated BMR [27], as expected for subjects who are seated rather than supine [28].

Because resting metabolic rate was measured in seated rather than supine Hadza subjects, some of whom were post-prandial, and because not all subjects were able to participate in RMR measurements, we used an estimate of basal metabolic rate (BMR) rather than measured RMR to calculate Hadza PAL. To test whether our comparisons of PAL for Westerners and Hadza were affected by the use of measured – rather than estimated – BMR values for Westerners, we reran these comparisons using estimated BMR for the Western subjects. Results were unchanged; Western men had lower PAL than Hadza men ($F(48)=9.08$, $p=0.004$) and Western women had lower PAL than Hadza women ($F(188)=5.40$, $p=0.02$).

Energy expenditure during walking was measured while walking on a flat outdoor track constructed near camp. Subjects wore their normal clothing and sandals and walked 700 to 800 meters at each of three different speeds ("slow", "normal", "fast") while energy expenditure was measured using breath-by-breath respirometry (Cosmed K4b2). A researcher (HP) walked with each subject throughout to ensure constant walking speed, and pace was checked every 100m using a stopwatch. Gross walking cost (kCal/min) was calculated for each speed by taking the average rate of energy expenditure measured over the final 200 to 400 meters. RMR was then subtracted from gross walking cost to calculate net walking cost (kCal/min), which was then divided by speed and body mass to give net cost of transport, COT (kCal kg^{-1} $meter^{-1}$). Minimum COT, COT_{min}, values were used for comparison with Western populations [28]. Mean COT_{min} for Hadza adults was 0.53 ±0.13 kCal kg^{-1} m^{-1} and is shown in Figure S1. Mean walking speed for COT_{min} trials was 1.2 ±0.07 meters/second. Mean COT_{min} was similar to that measured in Western populations [28].

To estimate the percentage of TEE spent on walking, mean COT_{min} was multiplied by mean daily travel distance for each subject, and this mean daily travel cost (kCal/day) was

divided by TEE. This provides an approximate, minimum cost of travel for each subject, since COT_{min} represents walking cost at the energetically optimal speed over level ground. Real-world travel for the Hadza involves walking at a range of speeds across varied terrain. Thus, the true proportion of TEE expended on travel is likely somewhat higher than these estimates.

Hadza Diet and Food Returns

As part of a long-term project on foraging returns, we identified and weighed all foods brought into each camp each day. Food weights were converted to calories using published estimates of energy density for Hadza foods [8,9]. For meat, energy density was estimated using a conservative value for domesticated animals (1100 kJ/100g dry weight), since game animals are relatively lean; sensitivity analyses revealed no effect on the estimated percentage of non-foraging foods when energy density values for meat ranged from 500 – 2000 kJ/100g dry weight. We are not aware of any published energy density values for Hadza game animals.

In Setako camp, food return data indicated that 3 – 4% of the calories consumed by residents came from traded agricultural foods (e.g., corn). In Sengeli camp, no traded foods were brought into camp during our two-week stay. Estimated percentages of calories consumed per food type are shown for each camp in Figure S2.

As noted in the main text, a significant percentage of calories in the Hadza diet come from honey. Food return data suggest honey accounted for 8% of caloric intake at Setako camp and 16% at Sengeli camp. These values, which are based on food brought back to camp, likely underestimate the true percentage of honey in the diet since honey is often eaten by men while out of camp on hunting forays.

Comparative U.S. TEE Data: New Measurements

Additional TEE measurements for U.S. adults (n=68) were conducted in free-living human subjects during 2-week periods using the DLW method [18]. Two baseline urine samples were collected, after which an oral dose of DLW was administered (0.20 g $H_2{}^{18}O$, 0.12 g ^2H_2O per kg total body water). Post-dose urine samples were collected on days 0, 7, and 14 and analyzed in duplicate for ^{18}O and ^2H abundance by isotope ratio mass spectrometry.
